# Association of *MTHFR* C677T variant genotype with serum folate and Vit B12 in Iranian patients with colorectal cancer or adenomatous polyps

**DOI:** 10.1186/s12920-021-01097-5

**Published:** 2021-10-13

**Authors:** Mahla Ghorbani, Marjan Azghandi, Reza Khayami, Javad Baharara, Mohammad Amin Kerachian

**Affiliations:** 1 Department of Biology, Faculty of Sciences, Mashhad Branch, Islamic Azad University, Mashhad, Iran; 2 Cancer Genetics Research Unit, Reza Radiotherapy and Oncology Center, Mashhad, Iran; 3 Department of Animal Science, Faculty of Agriculture, Ferdowsi University of Mashhad, Mashhad, Iran; 4 Medical Genetics Research Center, Mashhad University of Medical Sciences, Mashhad, Iran; 5 Department of Medical Genetics, Faculty of Medicine, Mashhad University of Medical Sciences, Mashhad, Iran; 6 Research Center for Animal Development Applied Biology and Biology Department, Mashhad Branch, Islamic Azad University, Mashhad, Iran

**Keywords:** Colorectal cancer, C677T, *MTHFR*, Folate, Vitamin B_12_, TaqMan

## Abstract

**Background:**

The incidence of colorectal cancer (CRC) has increased during recent years in Iran and other developing countries. Clinical studies suggest that essential folate dietary intake and moderate deficiency of methylenetetrahydrofolate reductase *(MTHFR*) may protect and reduce the risk of CRC. The present study aimed to investigate the clinical significance of C677T polymorphism within the *MTHFR* gene and its correlation with the serum folate and Vit B_12_ in the Iranian population suffering from CRC.

**Methods:**

Blood samples were taken from 1017 Iranian individuals (517 cases and 500 controls) who were referred for colonoscopy. TaqMan probe assay was performed for C677T *MTHFR* polymorphism. Sera were fractionated from the blood samples of 43 patients and controls and folate and Vit B_12_ concentrations were measured by a monobind kit. The correlation of *MTHFR* polymorphisms and folate/vitamin-B_12_ with CRC risk was analyzed.

**Results:**

In the current study, we found the frequency of three different genotypes of *MTHFR* polymorphism in the Iranian population i.e., CC, CT, and TT, to be 51.31, 26.73, 21.96 and 61, 32.2, 6.8 in case and control groups, respectively. The homozygote genotype of *MTHFR* rs1801133 polymorphism is associated with an increased risk of CRC by 3.68, 1.42, and 3.74-fold in codominant, dominant, and recessive models respectively (p value < 0.01). Our study revealed that there was no significant difference between the amount of folate and Vit B12 in the case and control groups (p value > 0.05).

**Conclusions:**

This study revealed that there was no significant difference between the amount of folate and Vit B_12_ in the case and control groups. Furthermore, our results demonstrated a higher risk association for 677TT and 677TT + C677T genotypes of *MTHFR* compared with 677CC carriers among CRC patients.

## Background

Colorectal cancer (CRC) is the third most diagnosed cancer and the second leading cause of cancer death (about 1,900,000 new cases and about 935,000 deaths) in the world [1]. This cancer is a multifactorial disease, involving genetic, epigenetic, and environmental risk factors [2, 3]. Because of smoking and a Westernized diet (high intake of meat and fat), the incidence has also been raised in Iran during the recent decades [4–8].

It has been shown that the dietary Vit B_12_ alone or combined with other factors (e.g. folate), could affect different parts of the colon and rectal [9]. While folate is a vitamin B group involved in multiple biochemical processes, it acts as an important modulator of carcinogenesis.

Folic acid, the synthetic form of folate, is a pivotal nutrient in a one-carbon cycle, which has major roles in the synthesis of nucleotides and methylation reactions. Besides, the methylenetetrahydrofolate reductase (*MTHFR*) enzyme has crucial functions during synthesis, repair, and methylation of DNA, as well as a duty in circulating folate levels [10]. Several studies have reported the impact of folate intake on tumorigenesis by transforming the template of gene expression, which indicates an association of polymorphisms among folate metabolizing genes such as *MTHFR* and the establishment of CRC [11].

The *MTHFR* gene polymorphism is betided with single nucleotide variants within codon 677 in exon 4 (C to T or Ala to Val). The result of this variant is to encode a thermolabile enzyme with reduced activity causing a decreased plasma folate level [12–15]. The 677CC is the wild-type form of the *MTHFR* gene. The 677TT homozygous variant and the heterozygous CT genotype have less than 30% and 65% of the normal enzyme activity (wild type homozygous CC genotype), respectively [16].

In the current article, we studied the association between the risk of CRC and *MTHFR* C677T polymorphism and also investigated the correlation of the polymorphism with serum folate/Vit B12 concentrations in our patients.

## Methods

### Study population and samples

A total of 517 blood samples of colon disease patients (98 adenomas and 419 adenocarcinomas), as well as 500 normal blood samples, were collected from Reza Radiotherapy and Oncology Center (RRCO), Mashhad, Iran with the ethic committee approval of Mashhad University of Medical Sciences, Mashhad, Iran (grant# 961906). Informed written consent had been obtained from all participants in this study. The control group was selected from individuals who were referred for a check-up colonoscopy without any diseases in the colon and rectum. In clinical evaluations, they had no serious GI diseases. Inclusion criteria for the patient group included patients who had positive colonoscopy reports for CRC and no first-relative family history of cancer. For the control group, inclusion criteria included individuals who had negative colonoscopy reports of CRC or any other colorectal diseases with no first-relative family history of cancer. Exclusion criteria for the patient and control groups were to identify a secondary GI disease during the study.

### MTHFR C677T polymorphism analysis

*MTHFR* C677T polymorphism was detected in DNA extracted from whole blood by the use of real-time PCR (TaqMan® assay). The DNA was extracted from 300 ul of blood using the standard salting-out method [17]. Primers and probes were synthesized by Bioneer Company (Bioneer Corporation, South Korea). The sequences of the primer and probe were as follows: primer forward, 5′-TGACCTGAAGCACTTGAAGGAGAA-3′, primer reverse, 5′-GGAAGAATGTGTCAGCCTCAAAGA-3′, probe C, 5′-ATGAAATCGGCTCCCG-3′ (reporter: FAM), probe T, 5′-ATGAAATCGACTCCCG-3′ (reporter Cy5).

### Folate and vitamin B12 measurement

Serum folate and vitamin B_12_ measurements were a total of 43 samples in the case and control groups. For this reason, 5 mL of blood was collected, and the serum was obtained by a centrifugation method. Serum was then stored frozen at − 80 °C until the time of usage. The determination of serum folate/vitamin B12 was done using ACCUBIND ELISA folate/Vit B12 test system kits (Monobind Inc., Lake Forest, CA 92630, USA).

### Statistical analysis

With the power of 85% and a significance level, prevalence, disease allele frequency, and genotype relative risk (RR) of 0.05, 0.0075, 0.23, and 1.38, respectively a sample size of 419 in the case group and 500 in the control group were calculated.

Association analyses were performed using SNPassoc [18]. The Hardy–Weinberg equilibrium (HWE) and the p-value for categorical variables were calculated by chi-square test. Mann–Whitney U test was used to establish the difference in levels of folate and Vit B12.

## Results

The association between the risk of CRC and *MTHFR* C677T polymorphism and the correlation of the polymorphism with serum folate/Vit B12 concentrations in Iranian patients were investigated. The details of age, gender distribution, and demographic characteristics of samples were shown in Tables 1 and 2. Moreover, the percentage of genotypes in different groups; normal, adenoma and adenocarcinoma has been indicated in Tables 1 and 3. Table 3 shows that the control group follows the Hardy Weinberg equilibrium. Notably, CRC patients and controls demonstrated significantly different frequencies for the rs1801133 alleles. Allele T carriers have an 84% higher risk of CRC than allele C carriers (OR = 1.84, 95% CI 1.5–2.26, p = 5.31e−09).

**Table 1:** Descriptive table of demographic variables

	Control*	Case**	p-value
Age^a^			
< 45	229 (45.8%)	86 (20.5%)	< 0.001
≥ 45	271 (54.2%)	333 (79.5%)	
Sex			
Female	261 (52.2%)	200 (47.7%)	0.2
Male	239 (47.8%)	219 (52.3%)	
Addiction^b^			
No	387 (77.4%)	353 (84.2%)	0.0115
Yes	113 (22.6%)	66 (15.8%)	
Tumor location			
Colon		206 (49.2%)	
Rectosigmoid		213 (50.8%)	

^*^Number of controls: 500; **number of cases: 419; Chi-squared test was used to determine the p-value; ^a^a cut off of 45 years old was determined for age according to American Cancer Society Guidelines and US Preventive Services Task Force Recommendation Statement [19, 20]; ^b^type of addiction = smoking (cigarette, hookah, opium)

**Table 2:** *MTHFR* 677 T polymorphism in adenoma samples

	N
*Sex*	
Female	48
Male	50
*Genotype*	
Tubular adenoma/CC	41
Tubular adenoma/CT	30
Tubular adenoma/TT	3
Tubulovillous adenoma/CC	4
Tubulovillous adenoma/CT	11
Tubulovillous adenoma/TT	5
Serrated adenoma/CC	1
Traditional serrated adenoma/CT	2
Villous/CC	1
*Location*	
Anal	1
Rectum	28
Sigmoid	46
Transverse colon	0
Descending colon	8
Ascending colon	12
Cecum	3
*Dysplasia*	
Low grade	80
High grade	18

Mean age = 57, max = 80, min = 27

**Table 3:** Allelic distribution and Hardy Weinberg equilibrium

Genotypes	Frequency (%)		
	Whole population	Controls	Cases
C/C	520 (56.58%)	305 (61%)	215 (51.31%)
C/T	273 (29.71%)	161 (32.2%)	112 (26.73%)
T/T	126 (13.71%)	34 (6.8%)	92 (21.96%)
*Alleles*			
C	1313 (71.44%)	771 (77.1%)	542 (64.68%)
T	525 (28.56%)	229 (22.9%)	296 (35.32%)
HWE (p value)	8.07E−16	0.0566881	2.93E−17

Table 4 represents the association analysis for each genetic model. Our data showed that homozygote TT carriers, compared with CC genotype, were associated with an increased risk of CRC both before and after adjustment for sex, age, and addiction (OR = 3.68; 95% CI 2.35–5.75, p = 2.516e−09). Besides, the recessive model showed that individuals with TT genotype had a higher risk of CRC than C allele carriers (CC + CT) (OR = 3.74 95% CI 2.42–5.78, p = 3.291e−10). Furthermore, the dominant model indicated that T allele carriers had a 42% higher risk of CRC after adjustment compared with CC homozygotes (OR = 1.42 95% CI 1.08–1.87, p = 1.134e−02).

**Table 4:** The association analysis for each genetic model

Model	Genotype	Control (%)	Case (%)	OR (CI95%)	P-value	OR^a^ (CI95%)	P-value^a^
Codominant	C/C	305 (61%)	215 (51.3%)	Ref		Ref	
	C/T	161 (32.2%)	112 (26.7%)	0.99 (0.73–1.33)	0.931	0.95 (0.70–1.29)	0.74361
	T/T	34 (6.8%)	92 (22%)	**3.84 (2.50–5.90)**	**9.01e−10**	**3.68 (2.35–5.75)**	**1.20e−08**
Dominant	C/C	305 (61%)	215 (51.3%)	Ref		Ref	
	C/T–T/T	195 (39%)	204 (48.7%)	**1.48 (1.14–1.93)**	**0.00322**	**1.42 (1.08–1.87)**	**0.0114**
Recessive	C/C–C/T	466 (93.2%)	327 (78%)	Ref		Ref	
	T/T	34 (6.8%)	92 (22%)	**3.86 (2.54–5.86)**	**2.48e−10**	**3.74 (2.42–5.78)**	**2.72e−09**
Over dominant	C/C–T/T	339 (67.8%)	307 (73.3%)	Ref		Ref	
	C/T	161 (32.2%)	112 (26.7%)	0.77 (0.58–1.02)	0.071	0.74 (0.55–1.00)	0.0528
log-Additive	0, 1, 2	500 (54.4%)	419 (45.6%)	**1.62 (1.35–1.95)**	**1.842e−07**	**1.58 (1.30–1.91)**	**2.353e−06**

Bold indicates a p-value less than 0.05 is statistically significant

^a^Adjusted for age, sex, and addiction

In subgroup analysis, TT genotype of rs1801133 polymorphism was associated with an increased risk in both colon and rectosigmoid cancer sites compared with CC homozygotes and C allele carriers (Table 5).

**Table 5:** The association analysis stratified for tumor location

Location	Model	Genotype	Control (%)	Case (%)	OR (95%CI)	P-value	OR^a^	P-value^a^
Colon	Codominant	C/C	305 (61%)	105 (51%)	Ref	**2.17e−05**	Ref	**5.30e−05**
		C/T	161 (32.2%)	62 (30.1%)	1.12 (0.77–1.62)		1.1 (0.76–1.6)	
		T/T	34 (6.8%)	39 (18.9%)	**3.33 (2–5.55)**		**3.27 (1.93–5.54)**	
	Dominant	C/C	305 (61%)	105 (51%)	Ref	**0.014404**	Ref	**0.021843**
		C/T–T/T	195 (39%)	101 (49%)	**1.5 (1.08–2.09)**		**1.48 (1.06–2.07)**	
	Recessive	C/C–C/T	466 (93.2%)	167 (81.1%)	Ref	**4.31e−06**	Ref	**1.04e−05**
		T/T	34 (6.8%)	39 (18.9%)	**3.2 (1.96–5.24)**		**3.16 (1.9–5.26)**	
	Over dominant	C/C–T/T	339 (67.8%)	144 (69.9%)	Ref	0.583813	Ref	0.558616
		C/T	161 (32.2%)	62 (30.1%)	0.91 (0.64–1.29)		0.9 (0.63–1.29)	
	log-Additive	0, 1, 2	500 (70.8%)	206 (29.2%)	**1.6 (1.26–2.02)**	**9.44e−05**	**1.57 (1.24–2)**	**0.000217**
Rectosigmoid	Codominant	C/C	305 (61%)	110 (51.6%)	Ref	**7.24e−10**	Ref	**5.88e−09**
		C/T	161 (32.2%)	50 (23.5%)	0.86 (0.59–1.27)		0.79 (0.53–1.18)	
		T/T	34 (6.8%)	53 (24.9%)	**4.32 (2.67–7)**		**4.16 (2.48–6.96)**	
	Dominant	C/C	305 (61%)	110 (51.6%)	Ref	0.020807	Ref	**0.080438**
		C/T–T/T	195 (39%)	103 (48.4%)	**1.46 (1.06–2.02)**		**1.35 (0.96–1.9)**	
	Recessive	C/C–C/T	466 (93.2%)	160 (75.1%)	Ref	1.17e−10	Ref	**1.49e−09**
		T/T	34 (6.8%)	53 (24.9%)	**4.54 (2.85–7.24)**		**4.5 (2.73–7.41)**	
	Over dominant	C/C–T/T	339 (67.8%)	163 (76.5%)	Ref	0.017874	Ref	0.006936
		C/T	161 (32.2%)	50 (23.5%)	0.65 (0.45–0.93)		0.59 (0.4–0.87)	
	log-Additive	0, 1, 2	500 (70.1%)	213 (29.9%)	**1.71 (1.37–2.14)**	**2.54e−06**	**1.64 (1.29–2.07)**	**3.75e−05**

Bold indicates a p-value less than 0.05 is statistically significant

^a^Adjusted for age, sex, and addiction

Tables 6 and 7 show the association of characteristic variables with genotypes. In Table 5, regardless of the genotype, patients 45 and over 45 years of age had a more pronounced risk effect than patients under 45. In this regard, patients with TT genotype represented the highest risk association (≥ 45 TT vs. < 45 TT: OR = 5.4 95% CI 2.21–13.19). In addition, participants with homozygote TT genotype and addiction were associated with a lower risk of cancer in comparison with TT carriers who did not have an addiction (addicted TT vs. not addicted TT, OR = 0.33 95% CI 0.13–0.81). Stratified analysis (Table 7) showed that within each gender group, the TT genotype was associated with a higher risk of CRC in comparison with CC carriers (female TT vs. female CC: OR = 4.89 95% CI 2.67–8.97; male TT vs. male CC: OR = 3.01 95% CI 1.63–5.57). Similarly, within each age group, homozygotes for the alternate allele showed an increased risk for both groups in comparison with CC genotype (< 45 TT vs. < 45 CC: OR = 2.31 95% CI 1.04–5.13; ≥ 45 TT vs. ≥ 45 CC: OR = 4.34 95% CI 2.49–7.56). Finally, in the no addiction group, TT genotype was also associated with increased risk of CRC in comparison with CC genotype of the same group (no addiction TT vs. no addiction CC: OR = 4.61 95% CI 2.76–7.69).

**Table 6:** The associations of the clinical characteristics within genotype

Genotype	Clinical feature	Characteristic	Control	Case	OR (95% CI)	P-value
C/C	Sex	Female	155	95	Ref	
		Male	150	120	1.31 (0.92–1.85)	0.136287
C/T		Female	89	54	Ref	
		Male	72	58	1.33 (0.82–2.15)	0.25070
T/T		Female	17	51	Ref	
		Male	17	41	0.8 (0.37–1.77)	0.587
C/C	Age	< 45	142	50	Ref	
		≥ 45	163	165	**2.87 (1.95–4.24)**	**9.77e−08**
C/T		< 45	71	23	Ref	
		≥ 45	90	89	**3.05 (1.75–5.31)**	**7.88e−05**
T/T		< 45	16	13	Ref	
		≥ 45	18	79	**5.4 (2.21–13.19)**	**0.000214**
C/C	Addiction	No	234	180	Ref	
		Yes	71	35	0.64 (0.41–1)	0.05210
C/T		No	131	95	Ref	
		Yes	30	17	0.78 (0.41–1.5)	0.4577
T/T		No	22	78	Ref	
		Yes	12	14	**0.33 (0.13–0.81)**	**0.016**

Bold indicates a p-value less than 0.05 is statistically significant

**Table 7:** The associations of the genotype within clinical characteristics

Clinical feature	Characteristic	Genotype	Control	Case	OR (95% CI)	P-value
Sex	Female	C/C	155	95	Ref	
		C/T	89	54	0.99 (0.65–1.5)	0.962720
		T/T	17	51	**4.89 (2.67–8.97)**	2.72e−07
	Male	C/C	150	120	Ref	
		C/T	72	58	1.01 (0.66–1.53)	0.97430
		T/T	17	41	3.01 (1.63–5.57)	0.00043
Age	< 45	C/C	142	50	Ref	
		C/T	71	23	0.92 (0.52–1.63)	0.7744
		T/T	16	13	**2.31 (1.04–5.13)**	0.0404
	≥ 45	C/C	163	165	Ref	
		C/T	90	89	0.98 (0.68–1.41)	0.900
		T/T	18	79	4.34 (2.49–7.56)	2.30e−07
Addiction^a^	No	C/C	234	180	Ref	
		C/T	131	95	0.94 (0.68–1.31)	0.72454
		T/T	22	78	**4.61 (2.76–7.69)**	4.76e−09
	Yes	C/C	71	35	Ref	
		C/T	30	17	1.15 (0.56–2.36)	0.704302
		T/T	12	14	2.37 (0.99–5.65)	0.052515

Bold indicates a p-value less than 0.05 is statistically significant

^a^Type of addiction = smoking (cigarette, hookah, opium)

**Fig. 1:**
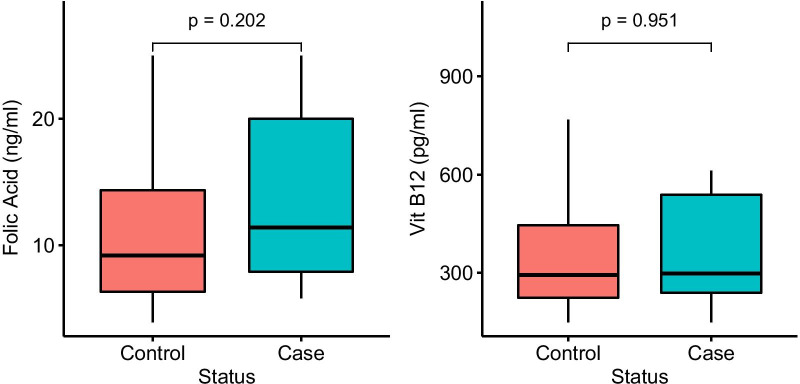
Comparison of folic acid and vitamin B12 levels between cases and controls. p-value was calculated using Mann–Whitney U test and p-value < 0.05 was defined as significant

The median concentration of serum Vit B12 was 298 and 293 pg/ml in cancer and normal groups, respectively. The median concentration of folic acid in cancer and normal groups was calculated at 11.4 and 9.2, respectively (Table 8). As illustrated in Fig. 1 and Table 8, there was no significant difference between the amount of folic acid and Vit B_12_ in cancer and normal groups (P = 0.202 and 0.951, respectively).

**Table 8:** Statistical analysis of folate and Vit B12 in adenocarcinoma and normal samples

Vit B12			Folic acid		
	Case (N = 19)	Control (N = 23)		Case (N = 17)	Control (N = 22)
High (> 982 pg/ml)	4	2	Normal (> 5.38 ng/ml)	17	19
Low (< 193 pg/ml)	2	4	Deficient (≤ 5.38 ng/ml)	0	3
Normal (193–982 pg/ml)	13	17			
X^2^	1.4993		X^2^	0.95805	
P	0.4725		P	0.3277	
Median (mad*)	298 (130.47)	293 (127.5)		11.4 (8.01)	9.2 (5.56)

*Median absolute deviation

## Discussion

In the present study, among CRC patients an increase in 677TT M*THFR* polymorphisms has been indicated.

Previous studies on the association of C677T polymorphism and susceptibility to CRC revealed no constant results. Some studies suggested a protective effect of TT genotype for colon cancer because a reduced risk of CRC progress was observed in TT individuals with a sufficient folate intake [19]. Chen et al. performed the first study to investigate the relation between the *MTHFR* C677T polymorphism and CRC. According to their results, the *MTHFR* C677T polymorphism affected enzyme activity and was involved in aberrant methylation and DNA synthesis, resulting in colorectal tumorigenesis [20]. Similar findings were subsequently reported by Slattery et al. [21], Ma et al. [22], and Le Marchand et al. [23]. However, due to statistically non-significant findings, several other published studies failed to support an impact of *MTHFR* gene polymorphisms on CRC risk [24–26].

It has been shown that the effect of 677TT *MTHFR* polymorphism, the phenotype of valine amino acid, significantly depends on folate intake. An *in-vitro* study on HCT116 colon carcinoma cells reported the association of valine amino acid (TT genotype) with increased genomic DNA methylation in an adequate folate level and a significantly lower DNA genomic methylation in folate deficiency suggested folate as a genotype modifier. Biochemical changes in the valine-containing enzyme are important, which shows the enzyme stabilization by the addition of 5-methyltetrahydrofolate (5-MTHF) to the culture medium. Therefore, folate might modify the correlation between SNPs and the CRC risk [27]. According to Kennedy et al., *MTHFR* 677TT genotype was linked to a lower incidence of CRC. Furthermore, the associated risk of CRC was decreased for both the *MTHFR* 677 CC and TT genotypes when total folate intake was high [28].

The migration and proliferation of cancer cells are two important events in cancer development. The main cause of death for cancer patients is metastasis, migration of cancerous cells from one organ to other organs. It has been demonstrated that about 10 µM folic acid reduced the migration and proliferation of human cell lines (COLO-205, LoVo, and HT-29) [29].

It has also been reported that the *MTHFR* 677TT genotype is one strong reason to lower the risk of proximal colon cancer. The site-specific analysis indicated the role of different molecular alterations in carcinogenesis in the proximal and distal of the colon and rectum. The more frequent genetic alterations in the distal side of the colon are *K-ras* and *P53* mutations but microsatellite instability (MSI) is more frequent in the proximal site in CRC [15, 30, 31]. Decreased risk of distal colon cancer, rectal cancer, and proximal colon cancer has been reported to be associated with the 677TT genotype [16]. However, in the present study, we found an increase of CRC risk in *MTHFR* TT and TT + CT genotypes compared to CC genotype (OR = 3.68 95% CI 2.35–5.75; OR = 1.42 95% CI 1.08–1.87, respectively). We also found that regardless of tumor site individuals with MTHFR 677TT genotype were associated with higher risk than C allele carriers (Colon: OR = 3.16 95% CI 1.9–5.26; Rectosigmoid: OR = 4.5 95% CI 2.73–7.41). Similarly, two studies on the Iranian population on 406 (175 cases, 231 controls) and 491 (234 cases, 257 controls) subjects, respectively reported that CC genotype has a protective effect on CRC [5, 32]. Although a more recent combined case–control study and meta-analysis on 2421 subjects have shown no significant association between *MTHFR* C677T polymorphism and the risk of CRC in the Iranian population suggesting the need for bigger sample size for *MTHFR* association studies [33]. There are also some reports indicating the increased risk of TT genotype in other populations [34, 35].

Some studies have reported a reduced risk of developing CRC with only TT genotype with a sufficient folate intake, suggesting a protective effect against CRC [21, 22]. In high folate intake cases, the risk of CRC risk is reduced for both *MTHFR* 677CC and 677TT genotypes [36]. Similarly, others have also reported the association of high-methyl diets like high folate dietary intake and low alcohol consumption with the protective effect of *MTHFR* 677TT genotype [16, 20, 23, 37, 38], although not everyone was able to demonstrate the protective effect of the *MTHFR* 677TT genotype even in high folate dietary intake [39–41]. However, it seems to be an association between the *MTHFR* polymorphism and dietary methyl supply, although the relationship remains inconsistent.

Even though we genotyped the MTHFR polymorphism in 1017 blood samples, there was still a limited sample size for folate and Vit B12 measurement that hindered us from incorporating this part of data into a comprehensive association study. Concerning the pilot study based on the limited number of samples for folate and Vit B12 measurement, further investigations are needed to support our findings. Furthermore, due to the heterogeneity of the Iranian population, there is an inevitable need for more multicentric studies in the Iranian population.

## Conclusions

In summary, our study revealed that there was no significant difference between the amount of folate and Vit B_12_ in the case and control groups. Furthermore, our results demonstrated a higher risk association for 677TT and 677TT + C677T genotypes of MTHFR compared with 677CC carriers among CRC patients in Iran.

## Data Availability

The datasets created during the current study are not publicly accessible due to the possibility of compromising the privacy of individuals. According to the written approval forms accepted by the Ethics Committee of the Mashhad University of Medical Sciences (MUMS), the data will only be available to researchers within project. The data could be available upon request from the corresponding author, Dr Mohammad Amin Kerachian (according to the MUMS rules and regulations).

## Abbreviations

MTHFR: Methylenetetrahydrofolate reductase
CRC: Colorectal cancer
SNP: Single nucleotide polymorphism
